# Identification of a novel role of IL-13Rα2 in human Glioblastoma multiforme: interleukin-13 mediates signal transduction through AP-1 pathway

**DOI:** 10.1186/s12967-018-1746-6

**Published:** 2018-12-20

**Authors:** Rukmini Bhardwaj, Akiko Suzuki, Pamela Leland, Bharat H. Joshi, Raj K. Puri

**Affiliations:** Division of Cellular and Gene Therapies (DCGT) Office of Tissues and Advanced Therapies (OTAT), Center for Biologics Evaluation and Research (CBER), Food and Drug Administration (FDA), Silver Spring, MD USA

**Keywords:** Glioblastoma, AP-1, Transcription factors, IL-13, IL-13Rα2

## Abstract

**Background:**

Previously, we have demonstrated that Interleukin 13 receptor alpha 2 (IL-13Rα2) is overexpressed in approximate 78% Glioblastoma multiforme (GBM) samples. We have also demonstrated that IL-13Rα2 can serve as a target for cancer immunotherapy in several pre-clinical and clinical studies. However, the significance of overexpression of IL-13Rα2 in GBM and astrocytoma and signaling through these receptors is not known. IL-13 can signal through IL-13R via JAK/STAT and AP-1 pathways in certain cell lines including some tumor cell lines. Herein, we have investigated a role of IL-13/IL-13Rα2 axis in signaling through AP-1 transcription factors in human glioma samples in situ.

**Methods:**

We examined the activation of AP-1 family of transcription factors (c-Jun, Fra-1, Jun-D, c-Fos, and Jun-B) after treating U251, A172 (IL-13Rα2 +ve) and T98G (IL-13Rα2 −ve) glioma cell lines with IL-13 by RT-qPCR, and immunocytochemistry (ICC). We also performed colorimetric ELISA based assay to determine AP-1 transcription factor activation in glioma cell lines. Furthermore, we examined the expression of AP-1 transcription factors in situ in GBM and astrocytoma specimens by multiplex-immunohistochemistry (IHC). Student t test and ANOVA were used for statistical analysis of the results.

**Results:**

We have demonstrated up-regulation of two AP-1 transcription factors (c-Jun and Fra-1) at mRNA and protein levels upon treatment with IL-13 in IL-13Rα2 positive but not in IL-13Rα2 negative glioma cell lines. Both transcription factors were also overexpressed in patient derived GBM specimens, however, in contrast to GBM cell lines, c-Fos is also overexpressed in patient derived specimens. Astrocytoma specimens showed lesser extent of immunostaining for IL-13Rα2 and three AP-1 factors compared to GBM specimens. By transcription factor activation assay, we demonstrated that AP-1 transcription factors (C-Jun and Fra-1) were activated upon treatment of IL-13Rα2 + GBM cell lines but not IL-13Rα2 − GBM cell line with IL-13. Our results demonstrate functional activity of AP-1 transcription factor in GBM cell lines in response to IL-13.

**Conclusions:**

These results indicate that IL-13/IL-13Rα2 axis can mediate signal transduction in situ via AP-1 pathway in GBM and astrocytoma and may serve as a new target for GBM immunotherapy.

**Electronic supplementary material:**

The online version of this article (10.1186/s12967-018-1746-6) contains supplementary material, which is available to authorized users.

## Background

Cancers of the brain and other nervous system components are amongst the top ten leading causes of cancer-related death among children, adolescents and adults in the United States. According to American Cancer Society predictions for 2018, approximately 23,880 new cases will be diagnosed and ~ 16,830 will succumb to this disease [1]. Several cell surface proteins expressed by brain tumor cells such as fibroblast growth factor receptor-1β, epidermal growth factor receptor, urokinase-type plasminogen activator receptor (uPAR) and transferrin receptor [2–5] have been identified as targets for novel therapies. Previously, we have demonstrated overexpression of interleukin 4 (IL-4) and IL-13 receptors on adult and pediatric brain tumors and meningiomas [6–10]. In contrast, normal human brain expresses barely detectable levels of IL-4R or IL-13R [9]. The differential expression of certain receptors between normal and malignant brain tissue may identify important biologic processes in cancer development and progression. Furthermore, proteins that are overexpressed or expressed selectively on cancer cells could be used as targets for therapeutic agents including fusion immunotoxins [11–17]. We have reported that IL-13Rα2 is overexpressed in ~ 78% of human GBM specimens. IL-13Rα2 is one of the two chains of IL-13R complex and is high affinity IL-13 binding receptor. IL-13 also binds to another chain IL-13Rα1 with low affinity and then forms a high affinity heterodimer with IL-4Rα and together mediates signal transduction through not only IL-13 receptors but also through IL-4 receptors. In solid tumor cells, both IL-4 and IL-13 phosphorylate different JAK kinases through IL-4Rα and IL-13Rα1, however, both cytokines phosphorylate and activate the same STAT6 protein [18–20]. In contrast, the IL-13Rα2 chain does not seem to signal through the STAT6 pathway. It inhibits signaling through the STAT6 pathway by both IL-13R and IL-4R [19–21]. We have shown that IL-13 could signal through IL-13Rα2 in a STAT6-independent, AP-1–dependent manner to induce activation of the TGFβ1 promoter resulting in inflammation and fibrosis in animals [22]. In addition, we have demonstrated that IL-13 can mediate signaling through IL-13Rα2 in human pancreatic and ovarian cancer cells [23]. In these models, IL-13 mediated cancer invasion and metastasis through IL-13Rα2 and signaling via AP-1/ERK pathway. Despite these studies, it is not known whether IL-13 can signal through IL-13Rα2 in human glioma tumors in situ and whether it utilizes AP-1 pathway.

AP-1 is a transcription factor that regulates a variety of target genes, leading to an increase in cell proliferation, invasion, and angiogenesis during tumor development [24]. AP-1 is not a single transcription factor but a series of related dimeric complexes of Fos and Jun family proteins. Fos proteins (c-Fos, FosB, Fra-1, Fra-2) form stable dimers with Jun proteins (c-Jun, JunB, JunD), whereas Jun proteins can form homodimers and heterodimers with Fos and activating transcription factor (ATF) proteins. Our previous studies on pancreatic ductal adenocarcinoma mouse model of human cancer suggested that IL-13 can mediate AP-1 signaling in IL-13Rα2 positive tumors. We also confirmed these observations in human ovarian and MonoMAc6 cell lines [22, 23, 25]. In addition, endothelin-1, a secretory growth factor secreted from malignant glioma cells, is also reported to increase AP-1 transcription factors and enhance migration of the glioma cells [26]. Wu et al. have identified and characterized a complex role of NFAT and AP-1 in expression of secreted from of IL-13Rα2 in GBM cells [27]. In the present study, we have examined whether IL-13 can signal through IL-13Rα2 via AP-1 pathway in GBM cell lines and in astrocytoma and GBM specimens in situ. We show that astrocytoma and GBM samples overexpress IL-13Rα2 and constitutively express AP-1 transcriptional factors. In contrast, IL-13Rα2 and AP-1 transcriptional factors are either absent or weakly expressed in normal human astrocytes and normal brain specimens.

## Methods

### Cell lines and tissue specimens

Human glioblastoma cell lines (U251, A172 and T98G) were obtained from the American Type Culture Collection (ATCC, Manassas, VA) and maintained in complete medium as per the instructions provided. Twenty-one human brain samples comprising of glioblastoma, astrocytoma and normal brain were obtained from Comprehensive Human Tissue Network (CHTN) after approval from U.S. FDA’s Research Involving Human Subjects Committee (RIHSC). These specimens included six normal brain, three astrocytomas and twelve glioblastoma samples. The histologic grade and demographic details were provided by the pathologists at CHTN along with the paraffin-embedded tissue sections (Table 1).

**Table 1:** Demography of Clinical specimens

Sample ID	Sex	Age	Clinical diagnosis
1	F	48	Glioblastoma multiforme
2	F	63	Glioblastoma multiforme
3	F	73	Glioblastoma multiforme
4	F	53	Glioblastoma multiforme
5	F	49	Glioblastoma multiforme
6	F	32	Glioblastoma multiforme
7	M	65	Glioblastoma multiforme
8	M	55	Glioblastoma multiforme
9	M	37	Glioblastoma multiforme
10	F	47	Glioblastoma multiforme
11	M	42	Glioblastoma multiforme
12	M	56	Glioblastoma multiforme
13	F	42	Astrocytoma
14	M	35	Astrocytoma
15	M	42	Astrocytoma
16	M	25	Normal brain
17	F	48	Normal brain
18	F	68	Normal brain
19	F	44	Normal brain
20	F	33	Normal brain
21	M	56	Normal brain

### Reagents and antibodies

IL-13 was purchased from PeproTech EC (Frederick Cancer Research Center, NCI, Frederick, MD). IL-2 was obtained from Chiron Corporation, Emeryville, CA. Anti-human IL13Rα2 polyclonal antibody from R&D, Minneapolis, MN., anti-IL-13 antibody from Santa Cruz, CA, USA, antibodies for AP-1 transcription factors (c-Jun, Fra-1, JunD, JunB and c-Fos) and transcription factor activation assay reagents were obtained from Abcam, Cambridge, MA. Polyclonal rabbit and goat serum was obtained from EMD Millipore, Billerica MA, USA. IL-13-PE, a chimeric fusion protein consisting of human IL-13 and a truncated form of *Pseudomonas* exotoxin (PE) was expressed and purified in our laboratory [28].

### RT-qPCR

Total RNA was isolated using RNA easy plus kit (Qiagen, Gaithersburg, MD) and reverse transcribed for performing RT-qPCR as described previously [9]. Gene sequence specific primers for AP-1 transcriptional factors and β actin were designed and synthesized at the CBER Core facility (Table 2) and the results were normalized and expressed as percent ratio of relative fluorescence units (RFU) of AP-1 gene and β-actin to evaluate the mRNA expression of AP-1 transcription factors. Each value is shown as mean ± SD of three independent experiments and expressed as % RFU of the controls.

**Table 2:** Primer sequences of AP-1 transcription factors for RT-qPCR analysis

1.	c-Jun	FW: GAAACGACCTTCTATGAC BW: GGTTACTGTAGCCATAAG
2.	Fra-1	FW: AGCAGAAGTTCCACCTGG BW: AATGAGGCTGTACCATCCA
3.	JunD	FW: AGCTCACAGTTCCTCTAC BW: TTCTGCTTGTGTAAATCC
4.	c-Fos	FW: AGACCAACTAGAAGATGAGAA BW: CTGCCAGGATGAACTCTA
5.	JunB	FW: TACCACGACGACTCATAC BW: GGTTTCAGGAGTTTGTAGT
6.	β-actin	FW: AAATCTGGCACCACACCTTC BW: GGGGTGTTGAAGGTCTCAAA

### Cell proliferation assay

GBM cells were plated in 96 well culture plates and incubated for 72 h in presence of different concentrations of IL-13 (0, 1.25, 2.5, 5, 10 and 20 ng/ml). Cell growth rate was monitored by MTS assay as previously described [29].

### Cytotoxicity assay

We tested the cytotoxic activity of IL-13-PE to confirm whether IL-13R expression in GBM cell lines are stable and able to internalize after binding to IL-13-PE. Cytotoxicity was determined by measuring radio-labeled leucin incorporation in a protein synthesis inhibition assay as reported previously [12, 28].

### ELISA

The secreted IL-13 protein from GBM cells was measured using Duoset ELISA development kit (R&D systems, MN) following the manufacturer’s instruction. Each experiment was repeated three times and values are shown as mean ± SD of three independent experiments.

### Activator Protein (AP-1) transcription factor activation assay

Two IL-13Rα2 positive (U251 and A172) and one IL-13Rα2 negative glioma cell line (T98G) were grown in complete medium and stimulated with 20 ng/ml IL-13 for 30 min when the tumor cells were in log phase. Nuclear proteins were isolated using the nuclear extraction kit following the manufacturer’s instructions (Thermo Fisher Scientific, Waltham, MA). 10 µg of total nuclear protein was used to determine AP-1 family member activity using the AP-1 transcription factor assay kit as recommended by the manufacturer’s instructions (Abcam, Cambridge, MA). The assay was performed in quadruplicate and results were expressed in arbitrary units after measuring optical density at 450 nm within 5 min with a reference wavelength absorbance at 665 nm.

### ICC and IHC analysis

These  assays were performed as described previously [9].Twenty thousand cells were plated in 8 well glass chambered EZ slides (Millipore, Billerica, MA, USA) overnight at 37 °C in a CO_2_ incubator and treated with IL-13 at 20 ng/ml for 30 min, 120 min, 240 min and overnight. IL-2 was used as a negative control. Antibodies and staining protocol was similar to cells and tissue sections as indicated in the staining protocols. The extent of immunostaining was ascertained by evaluating the staining intensity and counting number of positive cells in a set of 200 total cells or number of positive fields in biopsy specimens to calculate % positive cells or fields after viewing the samples at 200X magnification.

## Statistical analysis

Statistical analysis was performed using the Student t test for comparison between two groups and ANOVA among more than 2 using Graph Pad Prism software (Graph Pad, San Diego, USA). The level of statistical significance was set at a *P* value of 0.05 or less.

## Results

### Expression and characteristics of IL-13Rα2 in GBM cell lines

Expression of IL-13Rα2 mRNA in glioma cell lines was determined by RT-qPCR. As expected and as shown in Additional file 1: Figure S1, IL-13Rα2 mRNA was significantly overexpressed (P ≤ 0.001) in two GBM cell lines (U251, and A172), while T98G cell line expressed very low level of mRNA for IL-13Rα2. PM-RCC, a renal cell carcinoma cell line served positive control showed high mRNA expression of IL-13Rα2.

Consistent with the expression of IL-13R and as reported previously, U251 and A172 cell lines were highly sensitive to the cytotoxic effect of IL-13-PE while T98G cells were not sensitive (data not shown). We replicated the cytotoxicity experiment to demonstrate that IL-13R in glioma cell lines are still functional and behave as expected, as the cell lines are known to drift from their characteristics. The effect of IL-13 on proliferation of three glioma cell lines was examined, which showed no effect on the growth of these cells.

### Analysis of IL-13 secreted by GBM cells by ELISA

We determined the total amount of IL-13 secreted by glioma cell lines. U251 and A172 cell lines secreted 2420 pg/ml, and 1130 pg/ml of IL-13, while T98G secreted 561 pg/ml (data not shown).

### IL-13 mediates activation of AP-1 transcription factors through IL-13Rα2 in GBM cell lines

**Fig. 1:**
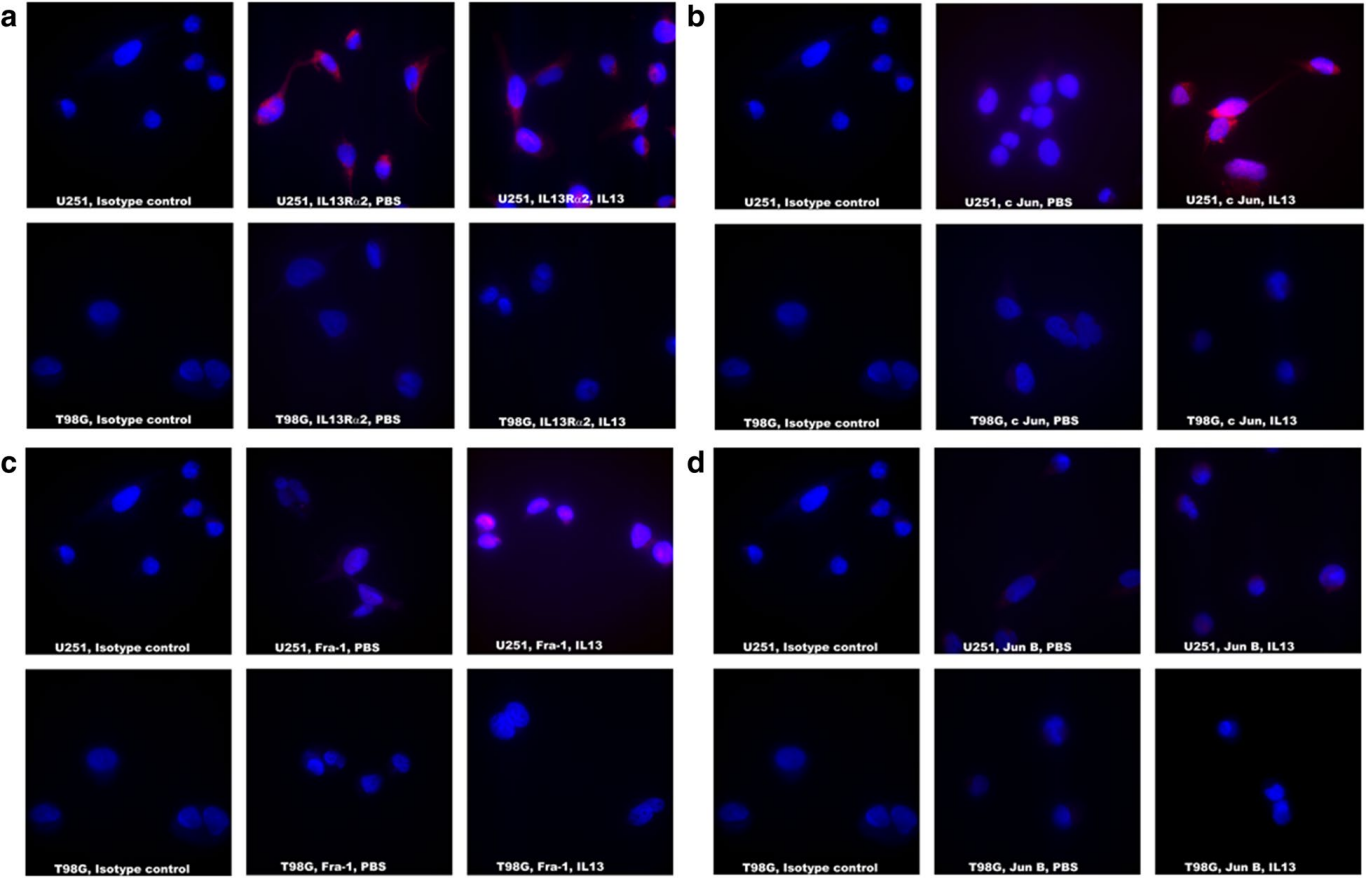
Evaluation of AP-1 transcription factors in IL-13 treated U251 and T98G cell lines. **a** IL13Rα2 staining with Streptavidin 594 (red) and DAPI used for nuclear staining (blue). **b** c Jun staining with Streptavidin 594 (red) and DAPI used for nuclear staining (blue). **c** Fra-1 staining with Streptavidin 594 (red) and DAPI used for nuclear staining (blue). **d** Jun B staining with Streptavidin 594 (red) and DAPI used for nuclear staining (blue). All images were captured at ×1000 magnification

We evaluated the extent of immunostaining of IL-13Rα2 and AP-1 transcription factors (c-Jun, Fra-1, JunD, c-Fos, and JunB) in three GBM cell lines. As shown in Fig. 1a–d, we observed surface staining for IL-13Rα2 and a brilliant nuclear staining for, c-Jun, Fra-1, and JunD only in IL-13Rα2 positive U251 GBM cells after incubation with IL-13. Table 3 summarizes the results of immune-staining for IL-13Rα2 and transcription factors in three glioma cell lines, which generally corroborated with RT-qPCR results for IL-13Rα2. U251 cells showed the highest (≥ 3 + immune-staining for c-Jun and Fra-1 with > 70% positive fields), while A172 showed the lower straining (2 + intensity and 50% positive fields). We also observed a varied degree of positive staining (1 + to 2 +) for JunD in receptor positive cell lines upon IL-13 treatment. JunD expression in U251 cells demonstrated an expression intensity of 2 + with < 50% positive cells, and A172 showed 1 + intensity with < 30% positive cells. c-Fos showed very low-level expression in U251 (2 +, < 20%) and A172 (> 1, < 10%) cell lines upon treatment with IL-13 (Table 3). JunB was not upregulated in both receptor positive cell lines (< 1, 0%) after treatment with IL-13. IL-13Rα2 negative T98G cells did not show upregulation of any of the AP-1 members. These results indicate that IL-13 upregulates varied degree of AP-1 transcription factors (c-Jun, Fra-1 and JunD) through IL-13Rα2.

**Table 3:** IFA staining analysis for AP-1 transcription factors in GBM cell lines

AP-1 transcription factors {extent of immunostaining (% positive cells)}												
	IL13Rα2		c-Jun		Fra-1		JunD		c-Fos		JunB	
	Control	+IL13	Control	+IL13	Control	+IL13	Control	+IL13	Control	+IL13	Control	+IL13
U251	3 +	3 + (> 70%)*	1.5+	3 + (> 70%)*	1 +	3.5 + (> 70%)*	1 +	2 + (< 50%)*	1 +	2 + (< 20%)*	< 1	< 1 (0%)*
A172	3 +	3 + (> 55%)*	< 1	2 + (40%)*	< 1	2 + (> 50%)*	< 1	1 + (< 30%)*	< 1	<1 (< 10%)*	< 1	< 1 (0%)*
T98G	< 1	< 1 (0%)*	< 1	< 1 (0%)*	< 1	<1 (0%)*	< 1	<1 (0%)*	< 1	<1 (0%)*	< 1	< 1 (0%)*

GBM cell lines were immunostained for extent of immunostaining for each phenotype. ≤ 1+, negative; 2 + , positive; 3 + , strongly positive; 4 + , more strongly positive. % cells are positive stained cells counted per total 100 cells (*)

We also performed a time course study by IL-13 treatment of GBM cell lines for four different time points (30 min, 120 min, 240 min and 16 h) (not shown). While IL-13Rα2 expression on GBM cell lines showed no change of expression pre and post IL13 treatment for 30 min, it produced optimal results for activation of AP-1 transcription factors. IL-2 incubation was used as a negative control, which did not show any immunostaining with anti-IL-13Rα2 antibody or anti-AP-1 transcription factor antibodies.

### RT-qPCR analysis for AP-1 transcription factor mRNA in GBM cell lines

**Fig. 2:**
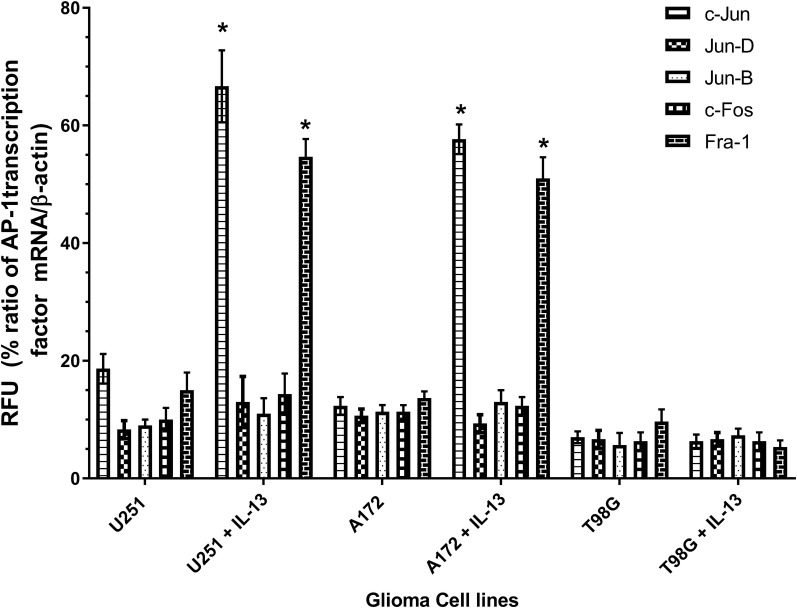
AP-1 transcription factors expression in GBM cell lines. mRNA was analyzed by RT-qPCR for AP-1 transcription factors expression in three GBM cell lines. Values are mean of three independent experiments each done in quadruplicate determinations. Results are expressed as % relative fluorescence units (RFU) normalized to β-actin expression

To confirm the upregulation of AP-1 transcription factors in IL-13Rα2 positive GBM tumor cell lines at molecular level, we performed RT-qPCR analysis to quantitate mRNA expression of all five AP-1 transcription factors in IL-13 treated GBM cell lines. As shown in Fig. 2, these results generally corroborated with ICC data, which demonstrated increased mRNA expression for c-Jun and Fra-1 in U251 and A172 GBM cell lines compared to corresponding untreated GBM cell lines (P = 0.0011). In contrast, IL-13Rα2 negative T98G cell line showed weak or no mRNA expression for these two transcription factors. Interestingly, mRNA levels of other three transcription factors such as c-Fos, Jun B and Jun D remained unaltered after IL-13 treatment in receptor positive and negative GBM cell lines.

### Analysis of AP-1 transcription factor activation

**Fig. 3:**
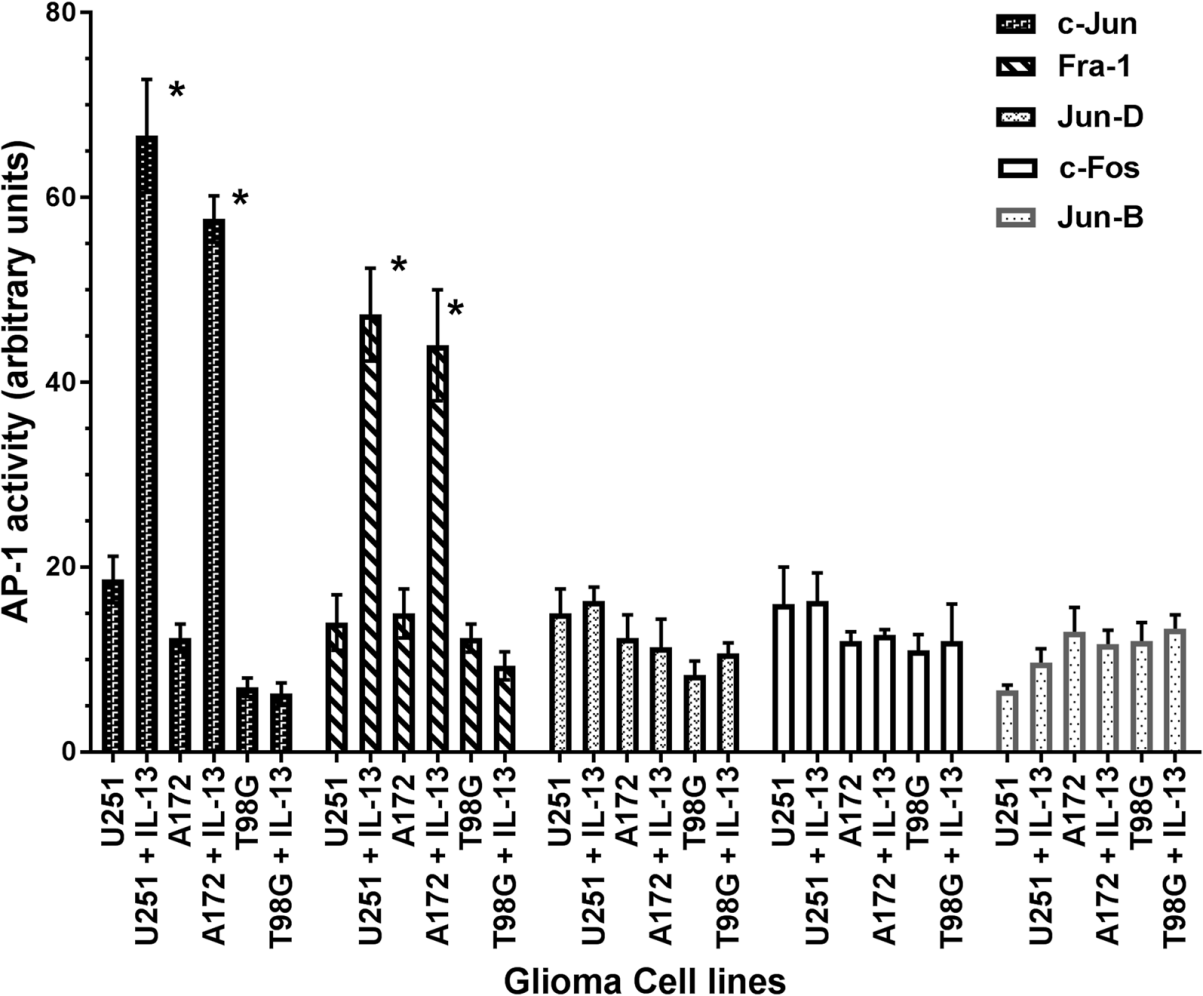
AP-1 transcription factor activation assay by ELISA. Nuclear extracts from two IL-13Rα2 positive GMB cell lines, U251 and A172 and IL-13Rα2 negative GBM cell line T98G were analyzed for AP-1 transcription factors after stimulation with IL-13. The assay was run in quadruplicate and results are expressed as arbitrary units after measuring the absorbance at 450 nm

We examined AP-1 transcription factor activation by performing a quantitative ELISA based assay using double stranded gene specific wild type and mutated oligonucleotide sequences of the CRE element (5′-TGACGTCA-3′-wild type) and a single base mutated sequence. The activated AP-1 transcription factor will bind to wild type CRE-element but not to mutated element. As shown in Fig. 3, c-Jun and Fra-1 transcription factors bound to nuclear fractions isolated from IL-13Rα2 positive IL-13 treated glioma cell lines (U251 and A172) compared to untreated cell lines at highly statistically significant level (P = 0.0018). However, there was no binding to mutated CRE element (data not shown). Consistent with the lack of expression of IL-13Rα2 in T98G cell line, IL-13 did not cause activation of c-Jun and Fra-1 transcription factors. Interestingly, consistent with the lack of or very low-level expression of other three AP-1 family members (Jun-B, c-fos and Jun-D), IL-13 did not activate binding to CRE elements in these GBM cell lines. These results demonstrate functional activity of AP-1 transcription factor in GBM cell lines in response to IL-13.

### Overexpression of IL-13Rα2 and AP-1 transcription factors in Astrocytoma and GBM specimens in situ

**Fig. 4:**
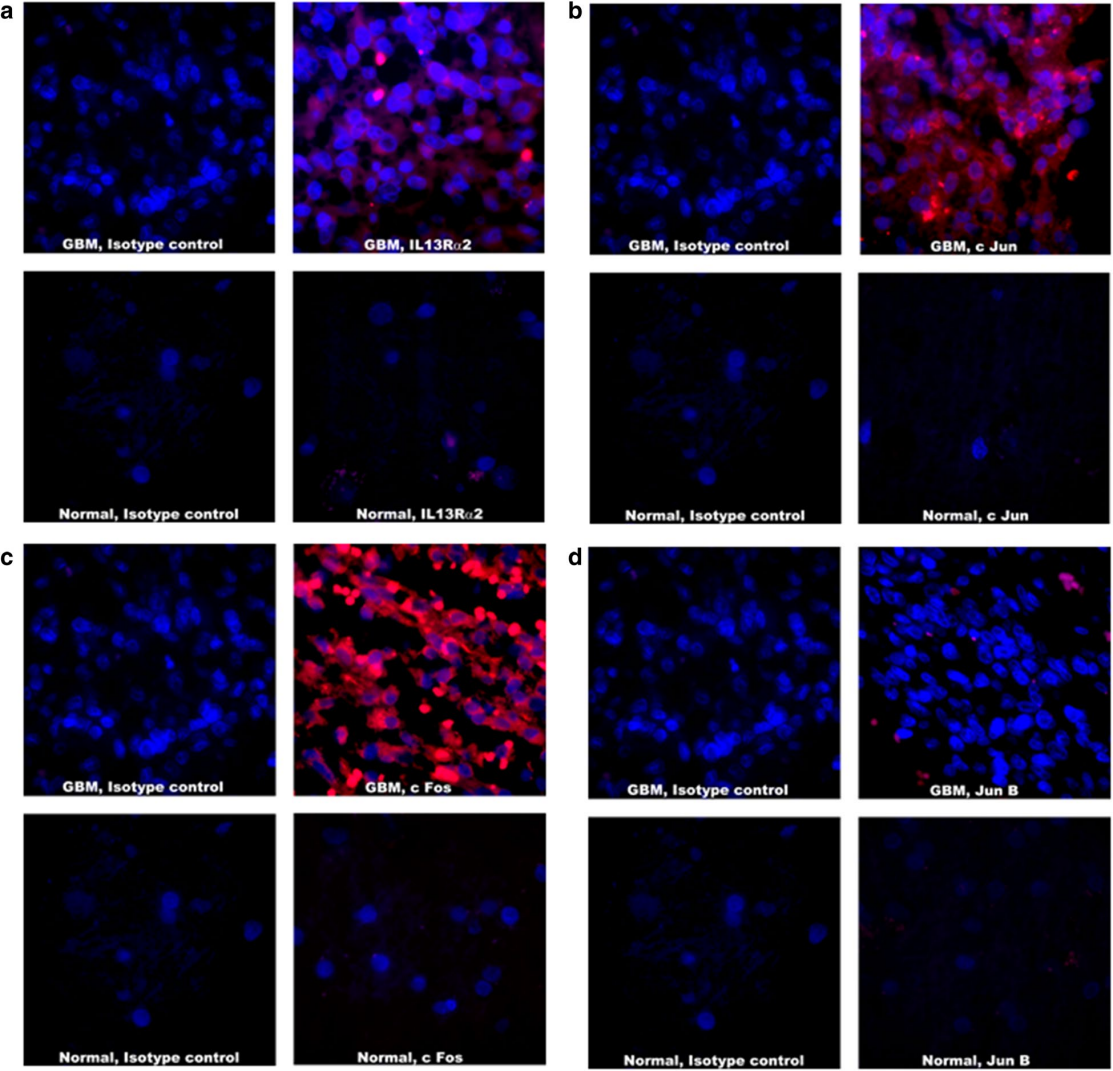
Analysis of AP-1 transcription factors in Human GBM samples and normal human brain sample. **a** IL13Rα2 staining with Streptavidin 594 (red) and DAPI used for nuclear staining (blue). **b** c Jun staining with Streptavidin 594 (red) and DAPI used for nuclear staining (blue). **c** c Fos staining with Streptavidin 594 (red) and DAPI used for nuclear staining (blue). **d** Jun B staining with Streptavidin 594 (red) and DAPI used for nuclear staining (blue). Immunostained sections were photographed at ×1000 magnification. Extent of immunostaining and percent positive field were counted and analyzed

**Fig. 5:**
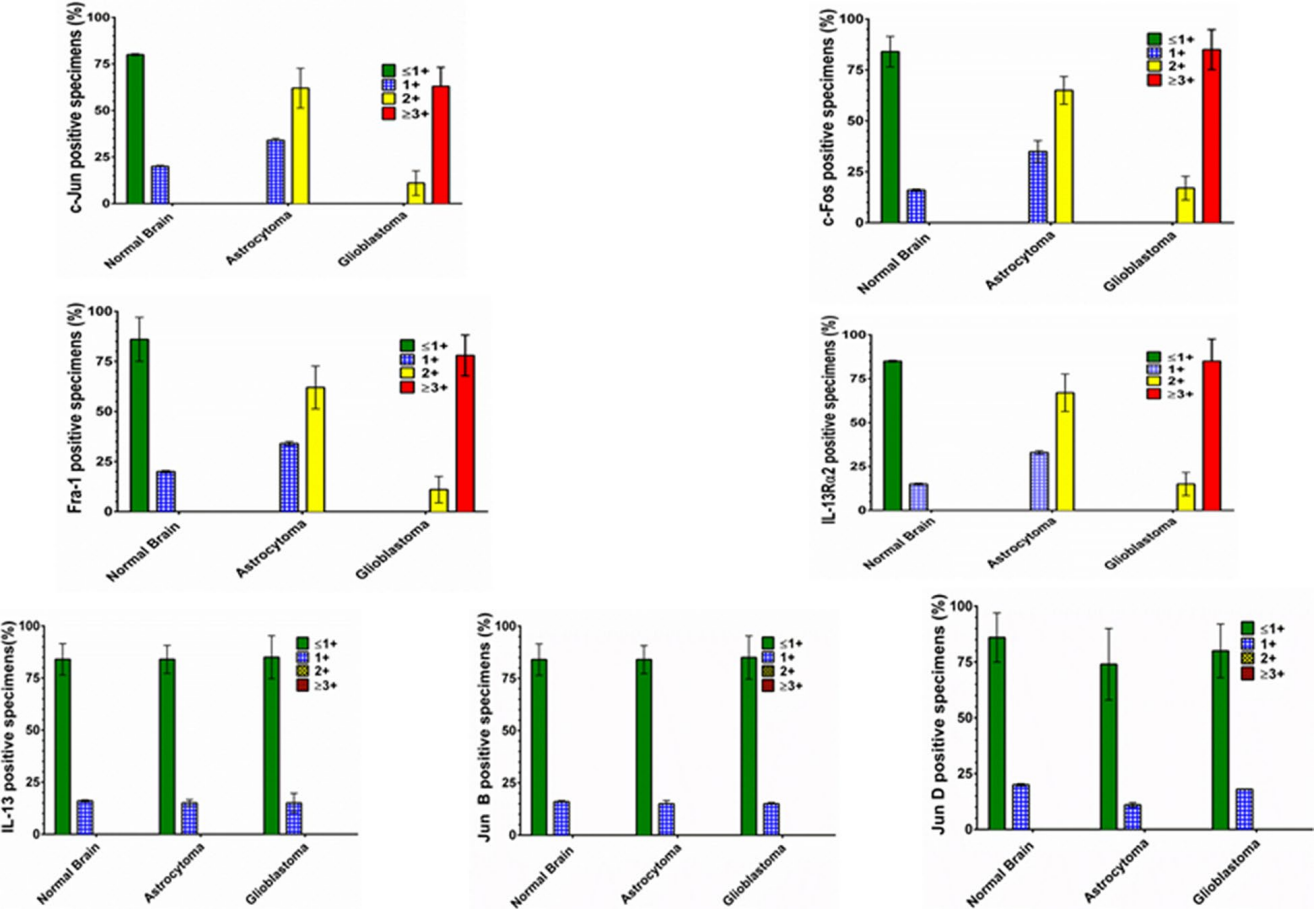
Evaluation of AP-1 transcription factors in human Glioblastoma, astrocytoma and normal brain specimens. Expression of AP-1 family members was analyzed in situ by IFA. The specimens were evaluated in a blinded manner by 3 investigators and the results are shown as mean ± SD. Extent of immunostaining for each phenotype corresponds to ≤ 1+, negative; 2 + , positive; 3 + , strongly positive; 4 + , more strongly positive

We evaluated the expression of AP-1 transcription factors by IHC to compare the extent of immunostaining and percent positive fields in GBM, astrocytoma and normal brain specimens. As shown in Figs. 4a–d and 5; twelve GBM specimens examined showed high degree of immunostaining for c-Fos, c-Jun and Fra-1 and a high percentage of positive fields. These specimens also showed strong immunostaining for IL-13Rα2 (3.5 +) in 74% positive stained cells (P < 0.001 compared to normal brain). Three astrocytoma specimens showed staining for IL-13Rα2 (1.5 + and 35% positive cells (P < 0.01) compared to normal brain), but the extent of staining and percent positive cells was lower than GBM (P < 0.001).

Similar to IL-13Rα2 expression, the extent of staining and percentage of positive fields for AP-1 transcription factors (c-Jun, Fra-1 and c-Fos) were highly statistically significant between GBM and normal brain (P < 0.001). The extent of immunostaining in GBM was highest for c-Fos (3.5 + , 74% fields) followed by Fra-1 (2.5 + , 73% fields), c-Jun (2.5 + , 56% fields) and JunD (1.5 + , 20% fields). JunB expression was the lowest among the AP-1 transcription factors (< 1+, 10% fields) in GBM specimens. We also observed that c-Jun and Fra-1 had similar extent of immunostaining (2.5 +) but the percent positive fields for Fra-1 were higher than that of c-Jun. Overall, these results suggest that AP-1 factors (c-Jun, Fra-1 and c-Fos) are overexpressed in the nuclei of patient derived GBM specimens.

Astrocytoma specimens showed lesser extent of immunostaining for the three AP-1 members (c-Jun, Fra-1 and c-Fos) compared to GBM (P ≤ 0.01). c-Fos showed 2 + staining and 42% positive fields followed by Fra-1 (2 + , 48% fields), c-Jun (1.5 + , 20% fields), and JunD (1 + , 18% fields). JunB staining intensity was < 1 + in only 8% fields. The expression of AP-1 factors between astrocytoma and normal brain specimen was statistically significant for Fra-1 (P < 0.001) and c-Fos (P < 0.01) but not for other members.

## Discussion

In the present study, we demonstrate that IL-13/IL-13Rα2 axis is important in mediating signal transduction by upregulating the AP-1 transcription factors in IL-13Rα2 positive, but not in IL-13Rα2 negative glioma cell lines. RT-qPCR analysis confirmed immunocytochemistry studies for the expression and upregulation of AP-1 transcription factors in GBM cell lines. Interestingly, there was a positive correlation between IL-13Rα2 surface expression and c-Jun, and Fra-1 upregulation at both mRNA and protein level in these GBM cell lines (U251 and A172). T98G glioma cells were considered IL-13Rα2 negative as these cells expressed extremely low levels of IL-13Rα2 mRNA, but no detectable surface expression by immunostaining. Because of the undetectable surface expression, we termed this cell line “IL-13Rα2 negative”. Consistent with the lack of IL-13Rα2 protein expression, IL-13 did not upregulate AP-1 transcription factors in T98G cell line. Neither mRNA nor proteins of the AP-1 transcription factors were upregulated after IL-13 treatment of IL-13Rα2 negative T98G glioma cell line.

Similar to in vitro results, we demonstrated that IL-13Rα2 are overexpressed in glioma surgical specimens in situ and AP-1 transcription factors are constitutively activated without exogenous administration of IL-13. Analysis of 21 GBM and astrocytoma and normal brain specimens revealed that all GBM specimens overexpressed IL-13Rα2 with high degree of correlation with c-Jun, Fra-1 and c-Fos activation while Jun D and Jun B were not expressed. c-Fos was the only additional factor overexpressed in patient derived GBM specimens, which was not seen in IL-13 treated receptor positive GBM cell lines. Astrocytoma samples showed lower expression of Fra-1 and c-Fos but none of the normal brain specimens were positive for IL-13Rα2 and AP-1 factors. Interestingly, these results demonstrate that different AP-1 transcriptional factors were upregulated in vitro in glioma cell lines and in situ in tissue sections. c-Jun and Fra-1 were only two transcription factors that were consistently induced by IL-13 in IL-13Rα2 positive glioma cell lines. In contrast, three members of AP-1 family, c-Fos, c-Jun and Fra-1, were highly expressed in glioma tissue samples in situ, while JunD and JunB were absent or weakly expressed. Low expression of c-Fos in GBM cell lines, despite being most conspicuous AP-1 family member may be attributed to some molecular or cellular or biochemical changes caused by immortalization of GBM cell lines. These data underscore importance of studying cell signaling mechanisms in tissue samples rather than relying solely on cell lines.

The mechanism of activation of AP-1 transcription factor in-situ is not clear. It is possible that IL-13 secreted by other cells can migrate to tumor bed and in turn activate AP-1 factors. IL-13 is produced by Th2 cells, mast cells, B cells, NK cells and granulocytes [14]. Since IL-13 is a secretory cytokine present in systemic circulation, the IL-13 in GBM tumor micro-environment may be sufficient enough to trigger AP-1 signaling. Alternatively, glioma tumor cells produce IL-13, which in turn caused autocrine activation of cell signaling through AP-1 pathway by engaging IL-13Rα2 chain. Our study demonstrated that the glioma cell lines produced only minute quantities of IL-13. Therefore, it is likely that IL-13 secreted by other cells in the systemic circulation may be involved in initiating signaling through AP-1 pathway in glioma tumors. Alternatively, growth factors or other factors present in the circulation may influence the expression of the AP-1 factors even in absence of IL-13 [30]. Based on these results, we speculate that autocrine and exocrine mechanism is operational in the activation of AP-1 transcription factors in GBM in vivo.

The function of AP-1 signaling through IL-13Ra2 was also confirmed by AP-1 transcription factor activation assay, which is a nuclear binding assay. These experiments demonstrated that 2 AP-1 transcription factors (c-Jun and Fra-1) were activated upon treatment of IL-13Rα2 positive GBM cell lines but not IL-13Rα2 negative cell line. These results demonstrate functional activity of AP-1 transcription factor in GBM cell lines in response to IL-13.

We have previously reported that IL-13Rα2 is a functional receptor as IL-13 mediates signaling in human pancreatic cancer cell lines. IL-13 induced TGF-β production via activation of AP-1 pathway [25]. We also demonstrated that IL-13/IL-13Rα2 axis is functional in mediating ERK1/2, AP-1 and matrix metalo-proteinase (MMP) activities only in IL-13Rα2-positive cells but not in IL-13Rα2-negative ovarian cancer cells [23]. IL-13 activated ERK1/2 signaling first followed by AP-1 activation through IL-13Rα2 in ovarian cancer cell lines, which in turn led to induction of MMP in IL-13Rα2 positive tumors. Other signaling pathways such as IRS1/2, PI3 K and AKT were not involved in IL-13 signaling in ovarian cancer cell lines. Based on published and our current results, it is possible that IL-13 may mediate ERK1/2 signaling leading to AP-1 activation in GBM, which is currently being investigated in our laboratory. Similar to our findings in vitro, other investigators have reported the signaling mediated by IL-13/IL-13Rα2 via AP-1 pathways in GBM and other tumor cell lines such as breast cancer, certain types of lung cancer cell line [31]. In the present study, we demonstrate for the first time that IL-13 can signal in situ in glioma samples rather than only in immortalized GBM cell lines (Additional file 2: Figure S2).

The significance of AP-1 signaling though IL-13Rα2 in glioma samples is not clearly understood. We have previously demonstrated that IL-13Rα2 is involved in cancer invasion and metastasis in pancreatic and ovarian cancers in animal models but not affects tumor growth [32]. In addition, we have shown that IL-13 activated AP-1 transcription factors in pancreatic cancer cell lines and ERK1/2 followed by AP-1 activation in ovarian cancer cell lines [23]. Since AP-1 family members are upregulated in astrocytoma and glioblastoma samples also, it is possible that IL-13Rα2 and AP-1 pathway may play a role in invasion, migration and infiltration of glioblastoma tumors within the intracranial cavity. Glioblastoma multiforme is an infiltrating disease. At the time of the diagnosis, 0.2% of glioma cells are already seeded in the distal part of the brain from the primary location [33]. Therefore, additional studies are needed to further delineate a role of IL-13Rα2 and AP-1 signal transduction pathway in glioma invasion and infiltration. Our preliminary results show that IL-13 can enhance cell invasion and migration of GBM cell lines in vitro (Joshi et al.; unpublished observations).

We also considered the role of IL-13Rα1 and IL-4Rα chains in IL-13 mediated signaling in GBM cell lines. GBM cell lines express IL-4Rα and IL-13Rα1 [9]. It is shown that IL-13 can signal through JAK/STAT-6 pathway in cells that express IL-4Rα and IL-13Rα1 chains [14, 21, 34]. But, in the current study, we did not see any activation of STAT-6 pathway, however, AP-1 members were activated. These studies supported our previous reconstitution studies, which demonstrated that IL-13 can signal through STAT-6 pathway when IL-13Rα1 and IL-4Rα chains are present. However, when IL-13Rα2 is also present, the STAT-6 activation is inhibited [35]. These results indicate that IL-13 mediates signaling through AP-1 pathway but not STAT-6 pathway in GBM cells that express IL-13Rα2.

We have previously demonstrated that IL-13Rα2 is an excellent target for various immunotherapy approaches such as IL-13 immunotoxin, IL-13Rα2 cDNA vaccine and combination of both which mediates significant efficacy and increased overall survival in animal models of human cancer. We have also demonstrated that IL-13Rα2 is directly involved in cancer invasion and metastasis in pancreatic and ovarian cancers [23, 32, 36]. Knocking down IL-13Rα2 significantly decreased invasion and metastasis and improved the survival of animals (Joshi et al. unpublished data). Since AP-1 is critical in signaling through IL-13Rα2, we believe that in addition to targeting IL-13Rα2, targeting of AP-1 may be an additional approach to immunotherapy of cancer where IL-13Rα2 and AP-1 play a critical role.

## Conclusions

**Fig. 6:**
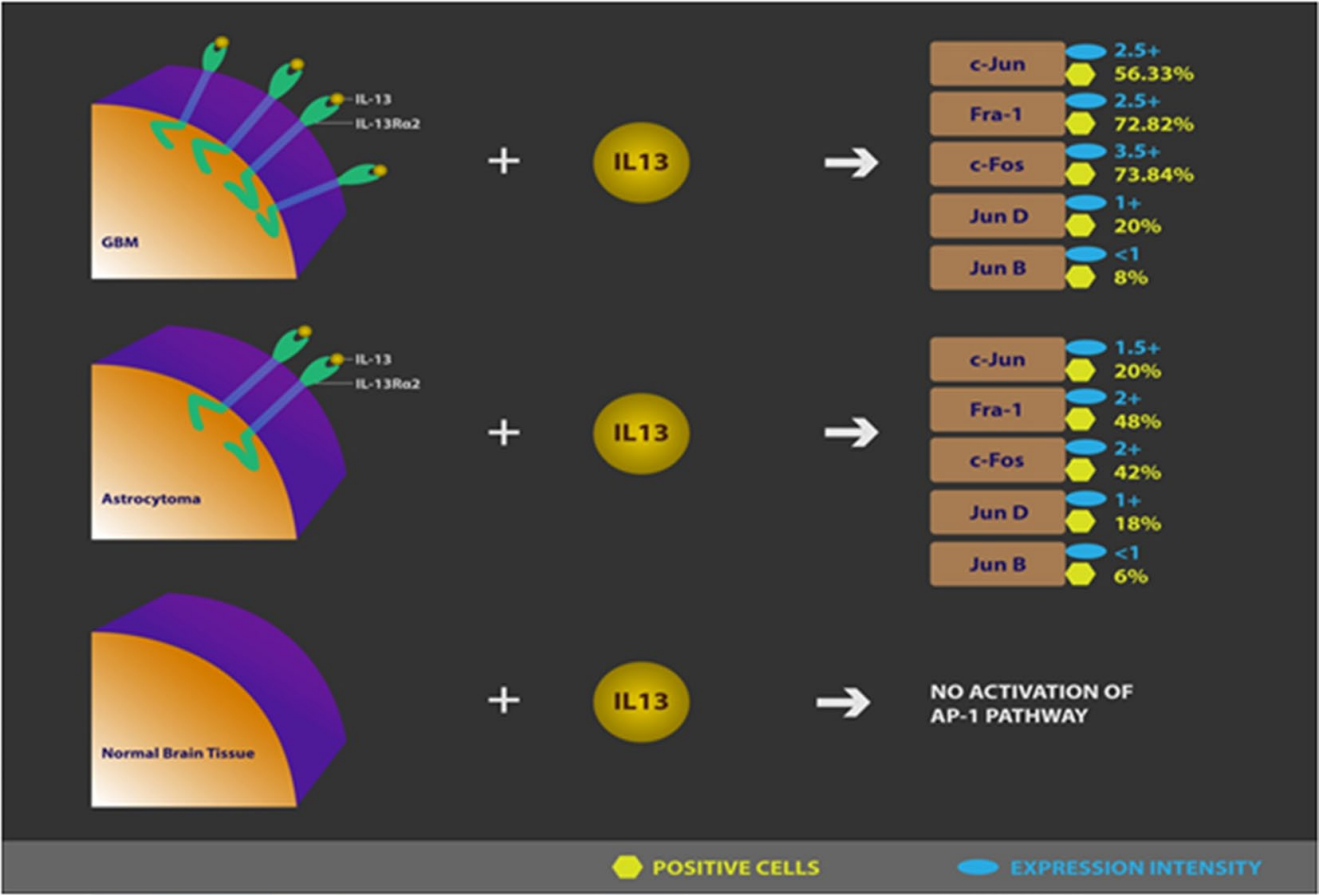
Analysis of AP-1 transcription factors in situ. Schematic representation of the results in human brain tissue specimen outlining the signaling mechanism of AP-1 in GBM, Astrocytoma and Normal brain tissues

In conclusion, since IL-13Rα2 is overexpressed in a variety of human primary tumors including astrocytoma and glioma as well as metastatic tumors, we predict that IL-13Rα2 targeted agents such as IL-13PE will not only target primary tumors but also metastatic tumors [37]. In addition, since IL-13/IL-13Rα2 axis is involved in AP-1 signal transduction pathway in human GBM similar to pancreatic and ovarian cancer (Fig. 6), we believe that specific targeting of this pathway may be an important target for therapeutic intervention for GBM therapy. Finally, since IL-13Rα2 and AP-1 pathway is involved in cancer invasion and metastasis, blocking this pathway may inhibit invasion, infiltration and metastasis of CNS cancers.

## Additional files

**Additional file 1: Figure S1.** IL-13Rα2 expression in GBM cell lines. mRNA was analyzed by RT-PCR for IL13Rα2 expression in four GBM and PM-RCC cell lines. Values are mean of triplicate determinations. Results are reported in relative fluorescence units normalized to β-actin expression. PM-RCC was used as a positive control. Values for IL13Rα2 expression are statistically significant from T98G (IL13Rα2 negative) cell line (*P < 0.001).

**Additional file 2: Figure S2.** Effect of IL-13 on cell proliferation of GBM cell lines- Cells (2 X 10^3^) in 100µl complete medium were plated per well in 96 well plates and incubated in presence of different concentrations of IL-13 for 48 h. The plates were incubated for additional 2 h after addition of MTS reagent and absorbance was measured at 490 nm.
