# Supplementary figures and images for: Identification of a novel role of IL-13Rα2 in human Glioblastoma multiforme: interleukin-13 mediates signal transduction through AP-1 pathway
Source: J Transl Med. 2018 Dec 20;16:369. doi: 10.1186/s12967-018-1746-6 (PMC6302477; doi:10.1186/s12967-018-1746-6)

**Supplementary figures:**

**Fig 1**

**
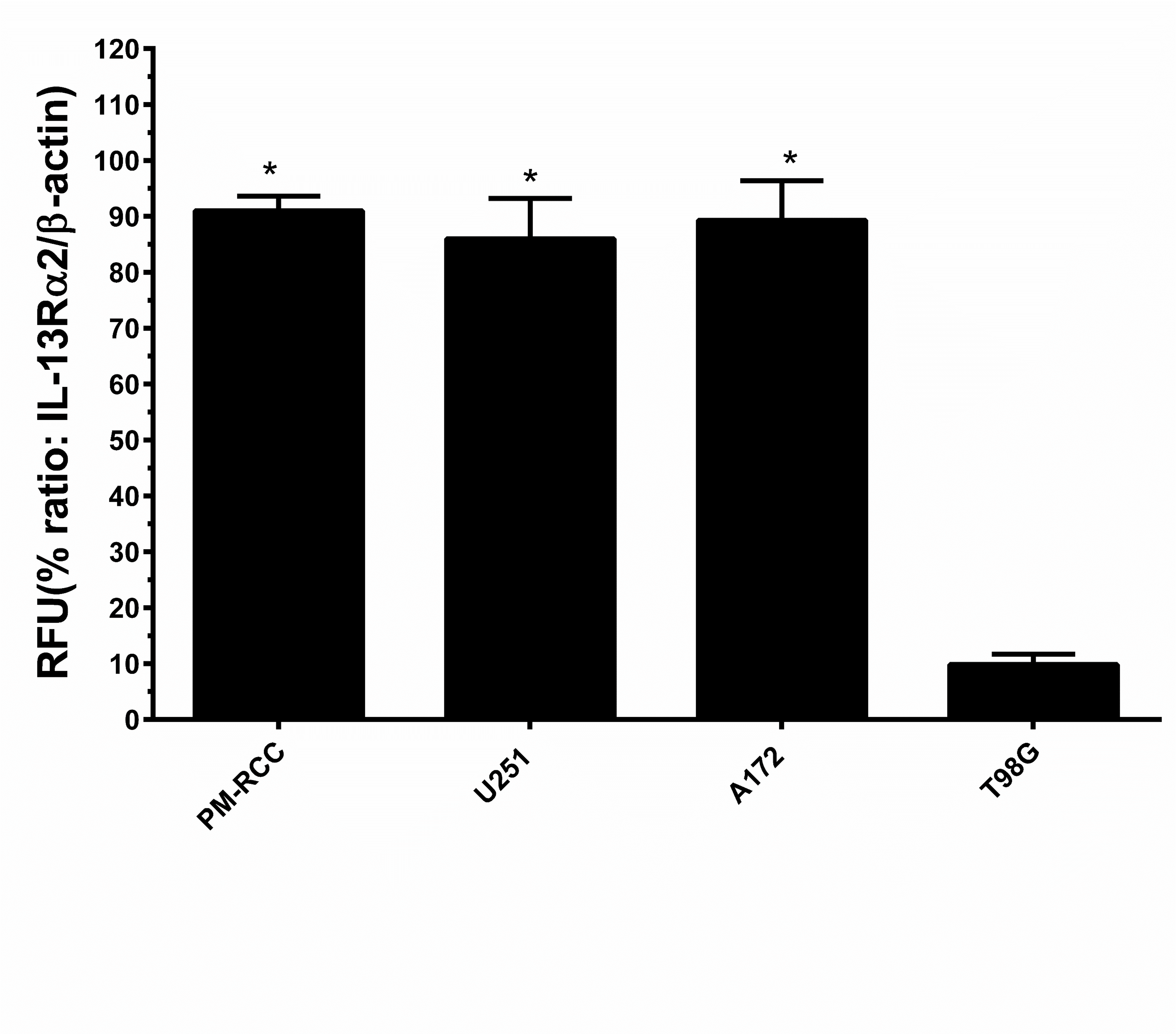
**

Supplement: Supplementary file 1 — Additional file 1: Figure S1. IL-13Rα2 expression in GBM cell lines. mRNA was analyzed by RT-PCR for IL13Rα2 expression in four GBM and PM-RCC cell lines. Values are mean of triplicate determinations. Results are reported in relative fluorescence units normalized to β-actin expression. PM-RCC was used as a positive control. Values for IL13Rα2 expression are statistically significant from T98G (IL13Rα2 negative) cell line (*P < 0.001). [file 12967_2018_1746_MOESM1_ESM.docx]

**Fig 2**


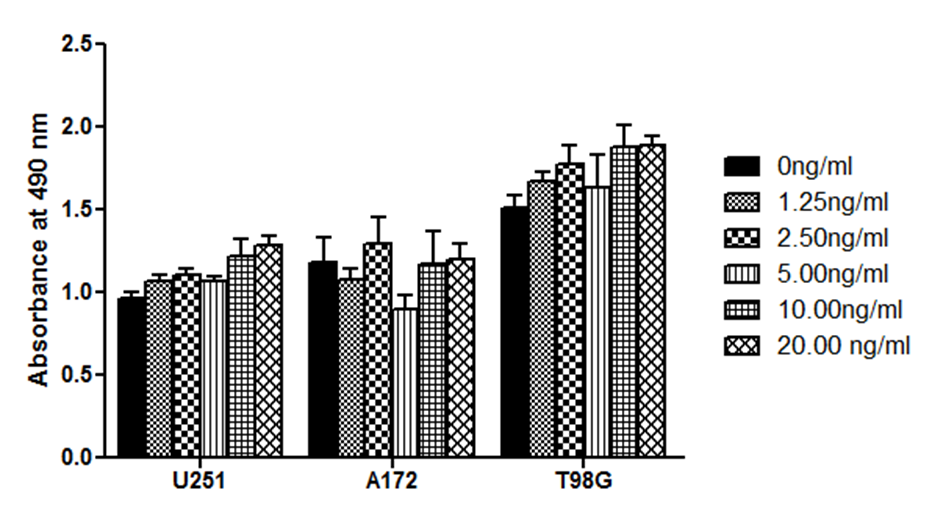

Supplement: Supplementary file 2 — Additional file 2: Figure S2. Effect of IL-13 on cell proliferation of GBM cell lines- Cells (2 X 103) in 100µl complete medium were plated per well in 96 well plates and incubated in presence of different concentrations of IL-13 for 48 h. The plates were incubated for additional 2 h after addition of MTS reagent and absorbance was measured at 490 nm. [file 12967_2018_1746_MOESM2_ESM.docx]
